# Cross-Phosphorylation, Signaling and Proliferative Functions of the Tyro3 and Axl Receptors in Rat2 Cells

**DOI:** 10.1371/journal.pone.0036800

**Published:** 2012-05-14

**Authors:** Jessica E. Brown, Meredith Krodel, Mauricio Pazos, Cary Lai, Anne L. Prieto

**Affiliations:** 1 Department of Psychological and Brain Sciences, Indiana University, Bloomington, Indiana, United States of America; 2 Medical College of Wisconsin, Milwaukee, Wisconsin, United States of America; Texas A&M University, United States of America

## Abstract

The dysregulation of receptor protein tyrosine kinase (RPTK) function can result in changes in cell proliferation, cell growth and metastasis leading to malignant transformation. Among RPTKs, the TAM receptor family composed of three members Tyro3, Axl, and Mer has been recognized to have a prominent role in cell transformation. In this study we analyzed the consequences of Tyro3 overexpression on cell proliferation, activation of signaling pathways and its functional interactions with Axl. Overexpression of Tyro3 in the Rat2 cell line that expresses Axl, but not Mer or Tyro3, resulted in a 5 fold increase in cell proliferation. This increase was partially blocked by inhibitors of the mitogen-activated protein kinase (MAPK) signaling pathway but not by inhibitors of the phosphatidylinositol 3-kinase (PI(3)K) signaling pathway. Consistent with these findings, an increase in ERK1/2 phosphorylation was detected with Tyro3 but not with Axl overexpression. In contrast, activation of Axl stimulated the PI(3)K pathway, which was mitigated by co-expression of Tyro3. The overexpression of Tyro3 enhanced Gas6-mediated Axl phosphorylation, which was not detected upon overexpression of a “kinase dead” form of Tyro3 (kdTyro3). In addition, the overexpression of Axl induced kdTyro3 phosphorylation. Co-immunoprecipitation experiments confirmed that the Axl and Tyro3 receptors are closely associated. These findings show that overexpression of Tyro3 in the presence of Axl promotes cell proliferation, and that co-expression of Axl and Tyro3 can affect the outcome of Gas6-initiated signaling. Furthermore, they demonstrate a functional interaction between the members of the TAM receptor family which can shed light on the molecular mechanisms underlying the functional consequences of TAM receptor activation in cell transformation, neural function, immune function, and reproductive function among others.

## Introduction

Cell proliferation is one of the basic cellular processes driving normal development, tissue repair and renewal. Receptor protein tyrosine kinases (RPTKs) are key regulators of proliferation and alteration of their function and that of their downstream targets can lead to malignant transformation [1], [2], [3], [4]. In this study we addressed the proliferative and signaling properties of the receptor Tyro3, and its ability to interact with its related receptor Axl. The TAM RPTK receptor family is composed of 3 structurally related members, Tyro3, Axl and Mer [5]. Two related proteins, protein S and Gas6, serve as ligands for the TAMs [6], [7]. Gas6 can bind and activate all three receptors, with binding affinities in the nM range [8], [9], [10], [11]. Functional studies have shown that the TAMs play an important role in the immune response by regulating the phagocytosis of apoptotic cells [12], the direct suppression of the inflammatory response [13], and the differentiation of natural killer cells [14]. In addition to their ability to regulate the immune response [15], these receptors have also been implicated in blood coagulation [16], [17], reproduction [18], [19], [20], diabetic nephropathy [21], and CNS function [22], [23], [24].

The 3 TAMs are upregulated in tumors of diverse origin and are frequently overexpressed in transformed cells [16], [25], [26]. The transforming potential of Tyro3 has been demonstrated by its ability to induce anchorage-independent growth on soft agar in fibroblastic cell lines and malignant melanoma cells [26], [27], [28], [29]. In addition, when injected into nude mice, Rat1b fibroblasts overexpressing Tyro3 stimulate tumor formation [28] and knockdown of Tyro3 in malignant melanoma cells decreases their proliferation [26]. Gas6 has been shown to induce cell proliferation via either Axl or Mer. However, it should be noted that in most of these studies the specific complement of TAMs expressed was not determined. For example, in NIH 3T3 cells, Gas6 signaling through Axl induced cell-cycle reentry via the activation of phosphatidylinositol 3-kinase PI(3)K and Src but a potential role for Tyro3 was not investigated [30], [31]. Gas6 has also been shown to elicit a proliferative response in rat vascular smooth muscle endothelia (VSMC) [32], [33], cardiac fibroblasts [34], mesangial cells [35], prostate cells [36] and Schwann cells [37]. *In vivo* studies also support a mitogenic role for Gas6 in tumors of diverse origin [38]. As Gas6 can activate all 3 TAMs, it is important to identify the complement of TAMs responsible for Gas6 mediated proliferation.

Cross-talk among cell surface receptors of several classes has been widely documented. In addition to forming homo- and heterodimers [4], RPTKs can be trans-activated by other receptor families such as G protein-coupled receptors (GPCRs) [39]. Studies addressing the interaction of the TAMs with each other and other receptors have been limited. One study has provided evidence for the co-immunoprecipitation of Axl and Tyro3 in a neuronal cell line suggesting a close association between these receptors [20]. In addition Axl has been shown to co-precipitate with IFNAR1 [13]. Other studies using TAM knockout mice have supported the concept that the receptors functionally interact [40], [41], [42], and at least two studies using these mice have reported that the presence of one TAM affects the phosphorylation of another TAM [41], [42]. In order to understand the mechanisms driving the transforming potential of Tyro3, the least characterized member of the TAMs, we studied its proliferative and signaling properties in the presence of Axl. When Tyro3 is overexpressed in Rat2 cells, a cell line that expresses Axl but not Tyro3 or Mer, Gas6 activation led to an increase in cell proliferation. This effect was mediated in part by activation of the mitogen-activated protein kinase (MAPK) signaling pathway but not influenced by the PI(3)K pathway. The co-expression of Tyro3 and Axl influenced the response to Gas6 as the introduction of Tyro3 led to a reduction in PI(3)K pathway signaling and an increase in MAPK pathway signaling. The overexpression of Tyro3 increased the tyrosine phosphorylation of Axl, while a kinase inactive form of Tyro3 lacked this ability. The cross-phosphorylation between Tyro3 and Axl along with their ability to co-precipitate suggest that they are closely associated. These findings also indicated that the co-expression of combinations of TAM receptors, in this case Tyro3 and Axl, can influence the outcome of Gas6-initiated signaling and lead to changes in cell function.

## Results

### Characterization of TAM Expression in Rat2 and Rat2/T3V5 Cells

**Figure 1:**
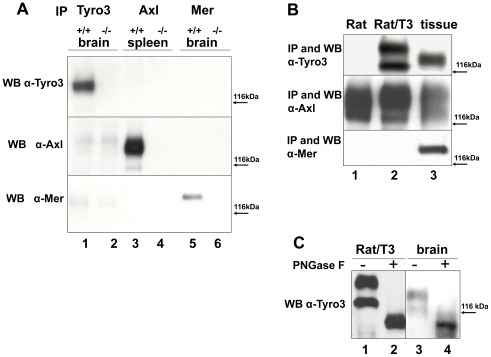
Characterization of anti-TAM specific antibodies and TAM expression in Rat2 cells. To determine whether the antibodies used to immunoprecipitate (IP) and detect Tyro3, Axl, and Mer “TAMS” by Western blotting were specific for each receptor, we used tissues derived from knockout mice for each of the TAMS. Whole brain and spleen detergent extracts were prepared from wild-type (wt) (^+/+^) C57BL/6 mice and from *tyro3* ^−/−^, *axl^−/−^*, and *mer* ^−/−^ knockout mice. As shown in **panel A**, After normalization for protein concentration, Tyro3 and Mer were IPed from brain detergent extracts (lanes 1–2 and 5–6 respectively) and Axl was IPed from spleen extracts (lanes 3–4). Tyro3 was IPed using α-FN2, Axl using #AF154 and Mer #AF591. SDS-PAGE was performed using 8% gels followed by Western blot analysis. The membranes were probed with α-Tyro3 (5424 serum 1∶3,000, **top panel),** affinity purified rabbit α-Axl (serum 1∶3,500, **center panel),** and rabbit α-Mer (1∶5,000 **bottom panel)**. These antibodies were used to characterize Tyro3, Axl, and Mer expression in Rat2 and Rat2/T3V5 cells as shown in **panel B.** Detergent cell extracts were prepared from untransfected Rat2 cells (**Rat, lane 1**), stably Tyro3 transfected Rat2/T3V5 cells (**Rat/T3 lane 2**), brain tissue extract (**lane 3, top and bottom panels**) or spleen tissue extract **(lane3, center panel).** After normalization of tissue extracts for protein concentration, Tyro3, Axl and Mer were immunoprecipitated (IPed) (see Fig. 1A for antibodies). SDS-PAGE was performed using 8% gels followed by Western blot analysis. The membranes were probed against Tyro3 **(top panel),** Axl **(center panel),** and Mer **(bottom panel),** as described in Fig. 1A. The observed differences in Tyro3 molecular weight depending on the source are due in part by N-linked glycosylation as shown in **panel C.** Detergent extracts were prepared from Rat2/T3V5 cells (**Rat/T3, lanes 1 and 2**), and adult rat brain tissue (**brain**, **lanes 3 and 4)**. Cell and tissue extracts were incubated overnight without (−) or with (+) PNGase F. SDS-PAGE was performed using 8% gels followed by Western blot analysis using α-Tyro3 antibodies.

We first characterized antibodies against individual TAM receptors to determine their specificity. Tyro3 and Mer immunoprecipitates were prepared from total brain extracts obtained from wild-type (wt), *tyro3* ^−/−^ and *mer* ^−/−^ knockout mice, while immunoprecipitates for Axl were prepared from wt and *axl* ^−/−^ knockout mouse spleens. Western blotting was performed with immunoprecipitates of Tyro3, Axl and Mer, using antibodies directed against each of these receptors. As shown in Fig. 1A (top panel), anti-Tyro3 antibodies only recognize Tyro3 in wt (^+/+^) extracts (lane 1) but not in those derived from the *tyro3^−/−^* mice (lane 2). Furthermore this antibody did not recognize proteins immunoprecipitated with the anti-Axl (lanes 3–4) and anti-Mer antibodies (lanes 5–6). Similarly, anti-Axl and anti-Mer antibodies only recognized bands corresponding to their cognate receptors (middle and bottom panels respectively). These results indicated that the antibodies utilized for immunoprecipitation and Western blotting are specific for each receptor of the TAM family.

As Gas6 is able to activate each of the 3 TAMs, we characterized the expression of Tyro3, Axl and Mer receptors in Rat2 cells and in a Rat2 cell line stably transfected with Tyro3 (Rat2/T3V5 cells). As shown in Fig. 1B, lane 1 untransfected Rat2 cells express Axl, but not Tyro3 or Mer, while the Rat2/T3V5 cells express both Tyro3 and Axl (lane 2). In the Rat2/T3V5 cells Tyro3 appears as a doublet (Fig. 1B, lane 2 top panel and Fig. 1C lane 1), although in brain Tyro3 appears mostly as a single band (Fig. 1A lane 1, Fig. 1B, lane 3 and Fig. 1C lane 3) [22], [27], [43]. In Rat2 cells both Tyro3 bands corresponded to N-linked glycosylated forms, as did the Tyro3 band present in brain. Treatment with N-Glycosidase F (PNGase F) resulted in molecular weight changes giving rise to a single Tyro3 immunoreactive band of approximately 95 kDa that was detected both in the Rat2 cells and in brain (Fig. 1C lanes 2 and 4).

### Gas6 Promotes the Proliferation of Rat2 Cells Transfected with Tyro3

**Figure 2:**
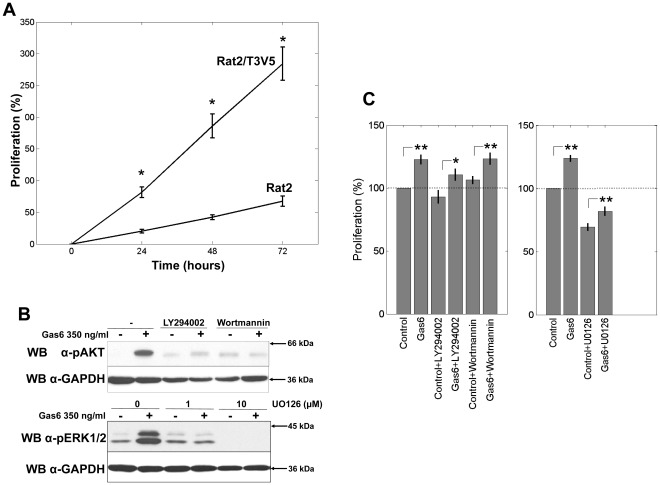
Gas6 induced cell proliferation of Rat2 and Rat2/T3V5 cells. Serum starved Rat2 and Rat2/T3V5 cells were stimulated with 250 ng/ml of Gas6 for 0–72 hrs (**panel A**). Proliferative activity is expressed as % increase over the optical density (OD) obtained at 0 hrs which was considered 0%. * = a significant increase in OD was observed in the Rat2/T3V5 cells when compared to Rat2 cells at 24, 48 and 72 hrs. p<0.01, two-sample t-test. Each experiment consisted of 4 wells for each Rat2 and Rat2/T3V5. All comparisons for n = 3 experiments. To determine the effectiveness of the signaling pathway inhibitors (**panel B**), Rat/T3V5 cells were incubated 45 min prior to activation with vehicle (DMSO), 1.5 µM LY294002, or 5.5 µM wortmannin **(top panel)** or with 1 µM or 10 µM U0126 **(bottom panel)**. The cells were activated with DMEM (-) or 350 ng/ml of Gas6 (+) for 20 min. Detergent cell extracts normalized for protein concentration were separated by SDS-PAGE using 4–20% gels. Western blotting was performed with α-pAKT **(top panel)**, and α-pERK1/2 **(bottom panel)**. The membranes were stripped and the blots were reprobed with α-GAPDH shown beneath each panel as protein loading control. These blots are representative of 3 experiments. To determine the effects of the signaling-pathway inhibitors on Gas6 mediated cell proliferation **(panel C)**, serum starved cells were stimulated with DMEM only (control) or 250 ng/ml of Gas6 for 72 hrs (Gas6) in the absence or presence of the indicated inhibitors. Proliferative activity is expressed as % increase of the optical density (OD). The OD obtained in the absence of addition of Gas6 (control) or inhibitors was considered 100%. Asterisks * denote  =  a significant increase in proliferation in the Gas6 treated cells relative to the untreated controls (*: p<0.05; **: p<0.01) two-sample t-test. The differences between Gas6 treated cells and their respective controls for vehicle only, LY294002 and wortmannin (left panel) were found to be the same at p>0.05. The differences between Gas6 treated cells and their controls for the two conditions, vehicle only, and U0126 (right panels) were different at p<0.01. Comparisons are based on n = 3 for the PI(3)K inhibitor panel (left) and n = 4 for the MAPK inhibitor panel (right).

We tested whether the introduction of Tyro3 would promote Gas6-mediated proliferation in Rat2 cells. Concentrations of Gas6 ranging from 0 to 500 ng/ml were evaluated, with a maximal effect achieved between 100 and 250 ng/ml (not shown). These concentrations are within those previously reported to induce Gas6-mediated proliferation [36]. As shown in Fig. 2A, treatment with 250 ng/ml of Gas6 induced a significant increase in cell proliferation of the Rat2/T3V5 cells compared to the Rat2 cells at all times points tested (24, 48 and 72 hrs). At 72 hrs, the increase in optical density was 5 times greater than that observed for the Rat2 cells.

In order to identify signaling candidates responsible for the proliferative effects of Gas6, we tested pharmacological inhibitors of molecules previously known to mediate Gas6 function. These included an inhibitor of the MEK kinase (U0126), a component of the MAPK signaling pathway, and two inhibitors of PI(3)K (wortmannin and LY294002). The PI(3)K and MEK inhibitors were first used to monitor the phosphorylation of AKT and ERK1/2, downstream targets of PI(3)K and MEK respectively. As shown in Fig. 2B, the inhibitors effectively blocked the Gas6-mediated activation of these two signaling pathways, as demonstrated by a reduction or elimination of AKT phosphorylation by wortmannin and LY294002 and a concentration-dependent reduction of ERK1/2 phosphorylation by U0126. When these inhibitors were tested in the cell proliferation assay (Fig. 2C), Gas6-induced cell proliferation as well as baseline proliferation in the absence of Gas6 were not significantly affected by wortmannin or LY294002. In contrast, treatment with the MEK inhibitor U0126 led to a 30% reduction in baseline proliferation of the Rat2/V5T3 cells in the absence of Gas6. In the presence of Gas6 the proliferation of MEK inhibitor treated cells increased compared to control, but only to half the extent of that observed in Gas6 stimulated cells. Gas6 could only partially overcome the inhibition of cell proliferation of Rat/T3V5 cells. These results suggested that the proliferative effect of Gas6 in Tyro3/Axl expressing Rat2/T3V5 cells was mediated in part through activation of the MAPK signaling pathway but not through activation of PI(3)K.

### Tyro3 Enhances ERK1/2 Signaling but Reduces Activation of PI(3)K Signaling

**Figure 3:**
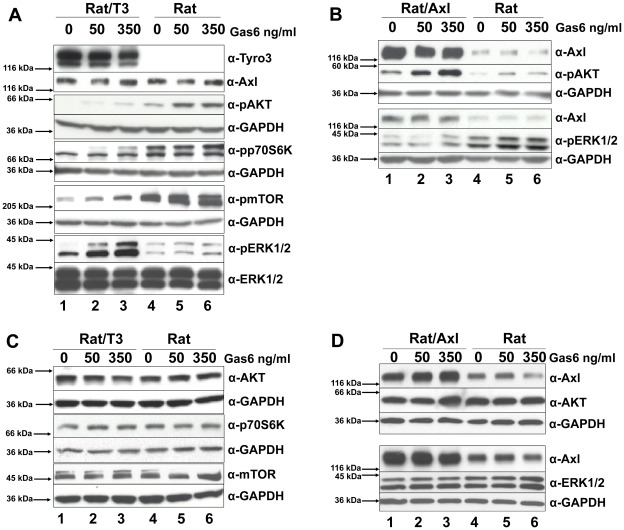
Tyro3 modulates MAPK and PI(3)K signaling pathways. As shown in **panel A**, Rat/T3V5 **(Rat/T3, lanes 1–3)** and Rat2 cells **(Rat, lanes 4–6)** were treated with 0, 50, and 350 ng/ml of Gas6 for 20 min. Detergent cell extracts normalized for protein concentration were separated by SDS-PAGE using 4–20% gels. Western blotting was performed with antibodies directed against α-Tyro3, α-Axl, α-pAKT, αpp70S6K, α-pmTOR, and α-pERK1/2. The membranes were stripped or cut and reprobed with α-ERK1/2 or α-GAPDH and shown beneath each panel as protein loading control. These blots are representative of 5 experiments. As shown in **panel B**, transiently transfected Rat2/Axl cells **(Rat/Axl, lanes 1–3)** and Rat2 cells **(Rat, lanes 4–6)** were treated and processed as above. Western blotting was performed with α-Axl, α-pAKT, and α-pERK1/2. The membranes were cut and reprobed with α-GAPDH shown beneath each panel as protein loading control. These blots are representative of 4 experiments. The total levels of MAPK and PI(3)K signaling pathway molecules was compared in Rat2 cells overexpressing Tyro3 **(panel C)** and Axl **(panel D).** For Tyro3 overexpressing cells **(panel C),** Rat/T3V5 cells **(Rat/T3, lanes 1–3),** and Rat2 untransfected cells **(Rat, lanes 4–6),** were treated with 0, 50 and 350 ng/ml of Gas6 for 20 min. Detergent cell extracts normalized for protein concentration were separated by SDS-PAGE using 4–20% gels. Western blotting was performed with antibodies directed against α-AKT, αp70S6K, α-mTOR. The membranes were cut and reprobed with α-GAPDH shown beneath each panel as protein loading control. For total levels of Tyro3, Axl, and ERK1/2 in Rat2 and Rat2/T3V5 cells, see **Fig. 3 A and B**. These blots are representative of 4 experiments. For Axl overexpressing cells **(panel D), t**ransiently transfected Rat2/Axl cells **(Rat/Axl, lanes 1–3)** and Rat2 untransfected cells **(Rat, lanes 4–6)** were treated and processed as above. Western blotting was performed with α-Axl, α-AKT, and α-ERK1/2. The membranes were cut and reprobed with α-GAPDH shown beneath each panel as protein loading control. These blots are representative of 4 experiments.

In order to further identify the signaling requirements for Tyro3-enhanced cell proliferation, we compared the activation of the PI(3)K and MAPK signaling pathways in Rat2/T3V5 cells expressing both Axl and Tyro3 (Fig. 3A, lanes 1–3) with the untransfected Rat2 cells that only express Axl (Fig. 3A, lanes 4–6).

As shown in Fig. 3A (lanes 4–6), Gas6 activation in Rat2 cells, which only express Axl, increased the phosphorylation of the downstream targets of PI(3)K, pAKT, pp70S6K and pmTOR in a concentration-dependent manner. Overexpression of Axl by transient transfection (Fig. 3B, lanes 1–3) also led to an increase in Gas6-mediated AKT phosphorylation. The increase in AKT/p70S6K/mTOR phosphorylation caused by Gas6 in the Rat2/T3V5 cells (Fig. 3A, lanes 1–3) was less than that observed in untransfected cells (Fig. 3A, lanes 4–6). The difference in phosphorylation of downstream PI(3)K targets in cells that only express Axl compared to cells expressing Axl/Tyro3 is not due to differences in overall levels of these molecules (Fig. 3C and 3D). In contrast, the phosphorylation of ERK1/2 was significantly enhanced by Gas6 activation in the Tyro3 transfected cells (Fig. 3A lanes 1–3), but not in the Axl overexpressing Rat2 cells lacking Tyro3 (Fig. 3B lanes 1–3). Interestingly, higher levels of pERK1/2 were detected in the untranfected cells, than in the Axl overexpressing cells, while the total levels of ERK1/2 were unchanged (Fig. 3D). These findings suggested that Tyro3 triggers ERK1/2 activation and attenuates the activation of the PI(3)K signaling pathway in Rat2/T3V5 cells. In addition, they demonstrated that in Rat2 cells Axl does not significantly activate ERK1/2, but does activate AKT, p70S6K and mTOR. The differences in phosphorylation in ERK1/2, AKT, p70S6K and mTOR observed in the presence of Axl and Tyro3 were not due to changes in their total levels as these remained were unaltered by overexpression of these receptors (see Fig. 3C and D).

### Tyro3 Increases Gas6-induced Axl Tyrosine Phosphorylation

**Figure 4:**
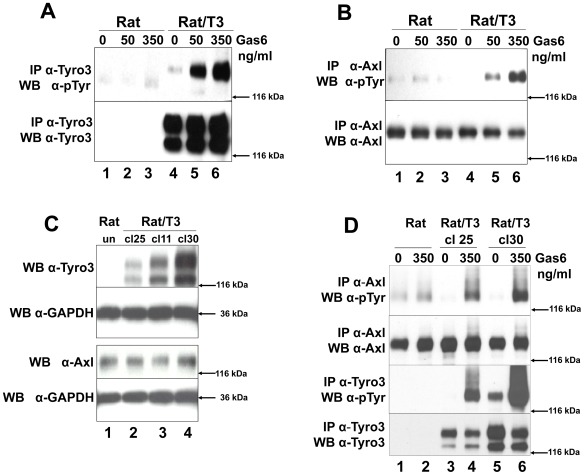
Tyro3 increases Gas6-induced Axl phosphorylation. Rat2 **(Rat lanes 1–3)** and Rat2/T3V5 cells **(Rat/T3, lanes 4–6)** were treated with 0, 50, 350 ng/ml Gas6 for 20 min. Detergent cell lysates were prepared and normalized for protein concentration. The samples were divided in two for Tyro3 **(panel A)** and Axl **(panel B)** immunoprecipitations (IP). This was followed by SDS-PAGE using 8% gels followed and Western blot analysis. The membranes were probed with anti-phosphotyrosine (α-pTyr) antibodies (PY20 and P99 mixture 1∶3,500) **(top, panels A and B).** The membranes were stripped and reprobed with α-Tyro3 serum 5424 (1∶3,500) **(A, bottom panel)** or affinity purified rabbit α-Axl (1∶3,500) **(B, bottom panel).** These blots are representative of 4 experiments. To determine whether Tyro3 expression affects Axl levels in Rat2 cells **(panel C),** detergent cell extracts were prepared from Rat2 cells **(lane 1)**, and independently derived stably transfected Rat2/T3V5 cell lines **(clone (cl) 25, lane 2; clone 11, lane 3; clone 30, lane 4)**. SDS-PAGE using 4–20% gels followed by Western blot analysis was performed on these extracts. The membranes were cut at the level of the 66 kDa marker and the top portion was probed with rabbit α-Tyro3 (5424 serum 1∶3,500) or rabbit α-Axl (1∶3,500). The bottom portion of the membranes were blotted with α-GAPDH (1∶500)**.** These blots are representative of 5 experiments. To determine if the levels of Axl phosphorylation depended on the levels of Tyro3 expressed **(panel D)** Rat2 cells **(lanes 1 and 2)** and Rat2/T3V5 cell lines cl25 **(lanes 3 and 4)** and cl30 **(lanes 5 and 6)** were activated with media only (0) or 350 ng/ml of Gas6 for 10 min. Detergent cell lysates were prepared and normalized for protein concentration. The samples were divided in two for Tyro3 and Axl immunoprecipitations (IP). SDS-PAGE using 8% gels followed by Western blot analysis was performed. The membranes were probed with anti-phosphotyrosine (α-pTyr) antibodies (PY20 and P99 mixture 1∶3,500) **(top and third panels).** An aliquot of the remaining Axl IPs were reloaded and probed with affinity purified rabbit α-Axl (1∶3,500) **(second panel from the top)**. The membrane corresponding to Tyro3 IP’s was stripped and reprobed with α-Tyro3 serum 5424 (1∶3,500) **(bottom panel)**. These blots are representative of 6 experiments.

We compared the ability of Gas6 to induce phosphorylation of Tyro3 (Fig. 4A) and Axl (Fig. 4B) in cells expressing Axl (Rat2) (lanes 1–3 both panels) and cells expressing both receptors (Rat2/T3V5) (lanes 4–6 both panels). Gas6 induced only a modest increase in phosphorylation of Axl in the untransfected cells (see Fig. 4B lanes 1–3 and Fig. 4D lanes 1 and 2), when compared to the higher Axl phosphorylation levels detected in the presence of Tyro3 (Fig. 4B lanes 4–6 and Fig. 4D lanes 3–6). To control for the possibility that higher levels of Axl phosphorylation were due to higher levels of its expression, we compared Axl levels across 3 different stably transfected Tyro3 clonal (cl) cell lines exhibiting different levels of Tyro3 expression (Fig. 4C, cl25 lane 2, cl11 lane 3, and cl30 lane 4 ). As shown in Fig. 4C no difference in total Axl levels were detected across the cell lines. Therefore changes in Axl phosphorylation in the presence of Tyro3 cannot be accounted for by changes in Axl expression as also indicated by the data in Fig. 3A. This suggests that increased levels of Axl phosphorylation are caused by differences in the levels of Tyro3 expression. To further test this possibility we used two of the clonal cell lines expressing different levels of Tyro3 described in Fig. 4C and tested whether increasing levels of Tyro3 would result in increased levels of Axl phosphorylation upon Gas6 stimulation. As shown in Fig. 4D, a small but detectable increase in Axl phosphorylation was observed in the absence of Tyro3 (Fig. 4D lanes 1 and 2), but the levels of Axl phosphorylation were much higher when Tyro3 was present (lanes 3–6). Furthermore they were proportional to the amount of Tyro3 present in the cells as observed when comparing the levels of Axl phosphorylation in the lower Tyro3 expressing clone (cl25 lanes 3 and 4) to the high Tyro3 expressing clone (cl30, lanes 5 and 6).

### Axl can Induce Phosphorylation of a Kinase Dead form of Tyro3, and both Receptors Co-immunoprecipitate

**Figure 5:**
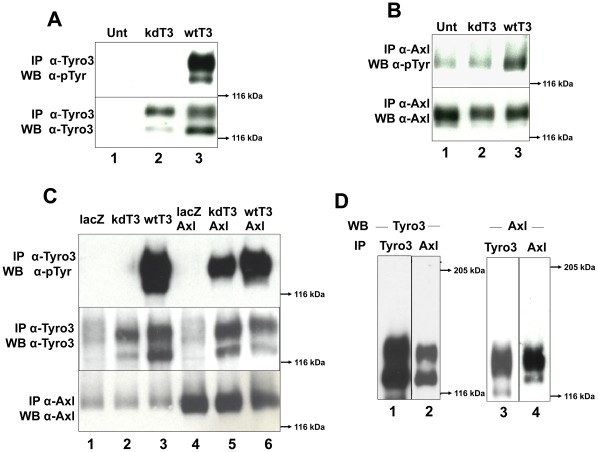
Axl cross-phosphorylates Tyro3 and both receptors co-immunoprecipitate. Wild-type and kinase dead forms of Tyro3 were tested for their ability to auto-phosphorylate **(panel A)** and phosphorylate Axl **(panel B).** Rat2 cells were transiently transfected kinase dead (kd)Tyro3 **(kdT3, lane 2)** or with wild-type (wt) Tyro3 **(wtT3, lane 3)**. The cells were activated with 350 ng/ml of Gas6 for 20 min. After protein normalization the extracts were divided in two, for Tyro3 immunoprecipitation (IP) **(panel A)** and for Axl IP **(panel B).** After SDS-PAGE using 8% gels and Western blotting, the membranes were probed with anti-phosphotyrosine (α-pTyr) antibodies (PY20 and P99 mixture 1∶3,500) **(top, panels A and B).** The membranes were stripped and reblotted with α-Tyro3 serum 5424 (1∶3,500, α-Tyro3) **(panel A bottom)** or re-probed with α-Axl (1∶3,500) **(panel B bottom).** These blots (**panels A and B**) are representative of 4 experiments. To determine if Axl can induce Tyro3 phosphorylation **(panel C)** Rat2 cells were transiently transfected with vectors encoding lacz **(lane 1)**, kdTyro3 **(kdT3, lane 2)**, wtTyro3 **(wtT3, lane 3)** or doubly transfected with lacz/Axl **(lane 4)**, kdTyro3/Axl **(kdT3/Axl,** **lane 5)** and wtTyro3/Axl **(wtT3/Axl, lane 6)**.The cells were activated with 350 ng/ml of Gas6 for 20 min. After protein normalization the extracts were divided in two, for Tyro3 immunoprecipitation (IP) **(top and center panels)** and for Axl IP **(bottom panel).** After SDS-PAGE in 8% gels and transfer, the membranes were probed with anti-phosphotyrosine (α-pTyr) (PY20 and P99 mixture 1∶3,500) antibodies **(top panel),** and rabbit α-Axl **(bottom panel).** The membrane probed with α-pTyr (Tyro3 IPs) was re-probed with α-Tyro3 (**center panel**). These blots are representative of 5 experiments. To determine whether Tyro3 and Axl co-immunoprecipitate, **(panel D)** Rat2/T3V5 cells were activated with 350 ng/ml Gas6 for 10 min. Detergent extracts were normalized and divided in two, for Tyro3 immunoprecipitation (IP) using α-FN2 Tyro3 antibodies **(IP Tyro3, lanes 1 and 3)** and for Axl IP using mouse monocolonal α-Axl antibodies **(IP Axl, lanes 2 and 4).** The samples were separated by SDS-PAGE using 6% gels and blotted with α-Tyro3 serum 5424 (1∶3,500) (**lanes 1 and 2)** or rabbit α-Axl antibodies (1∶3,500) **(lanes 3 and 4).** These blots are representative of 4 experiments.

In order to determine if the kinase activity of Tyro3 is required to induce Axl phosphorylation, we generated a kinase inactive or “kinase dead” construct (kdTyro3) by mutating K535 to M (K535M). This results in a receptor that is unable to undergo autophosphorylation [44], but that can still be trans-phosphorylated. As shown in Fig. 5A lane 2, we failed to detect tyrosine phosphorylation of the kdTyro3 mutant when activated by Gas6 in contrast to the sharp increases in phosphotyrosine levels observed in the wild-type (wt) Tyro3 construct (compare kdT3, Fig. 5 lane 2 with wtT3, lane 3). We then compared Axl phosphorylation levels (Fig. 5B) in Rat2 cells transiently transfected with either kdTyro3 (lane 2) or wtTyro3 (lane 3). As shown in Fig. 5B, Axl phosphorylation levels in untransfected cells (that do not express Tyro3) (lane 1), are comparable to those observed in cells transfected with the kdTyro3 construct (lane 2). In contrast, in cells expressing wtTyro3 (lane 3) the levels of phosphorylation of both Tyro3 and Axl are significantly higher than in the untransfected cells (Fig. 5, compare lane 3 with lane 2 in panels A and B). These results indicated that the increase in Axl phosphorylation observed upon Tyro3 overexpression is due to Tyro3 activation.

Kinase inactive RPTKs can be trans-phosphorylated by their catalytically active dimerization partners [45] or cross-phosphorylated by other kinases [39]. In the experiment shown in Fig. 5A lane 2, the phosphotyrosine levels of the kdTyro3 were not detectable despite the presence of Axl (panel B). This low or lack of phosphorylation may be attributed to the low endogenous levels of Axl, which may be insufficient to noticeably increase the levels of kdTyro3 phosphorylation. Indeed, even Axl itself is only weakly activated in these cells in response to Gas6 (see Fig. 4B lanes 1–3, Fig. 4D lanes 1–2 and Fig. 5B, lane 2). To determine if Axl is capable of trans-phosphorylating kdTyro3, we boosted the levels of Axl by co-transfecting Rat2 cells with Axl/kdTyro3 or Axl/wtTyro3. As shown in Fig. 5C, the increased expression of Axl (lanes 4–6) resulted in a notable elevation of kdTyro3 phosphorylation (lane 5) when compared to those expressing lower levels of Axl (lane 2). These results indicated that Tyro3 can be crosss-phosphorylated by Axl since the kdTyro3 lacks kinase activity. We then tested whether Tyro3 and Axl co-precipitated as an indication that the two receptors closely interact.

When Tyro3 immunoprecipitates were blotted with anti-Tyro3 (Fig. 5D lanes 1) monomeric forms of the approximately 125–120 kDa doublet characteristic of Tyro3 were detected. When the Tyro3 immunoprecipitates were blotted against Axl, we also detected monomeric Axl (Fig. 5D lane 3). When Axl immunoprecipitates were blotted against Axl we detected bands corresponding to monomeric Axl as expected (Fig. 5D lane 4). When these Axl immunoprecipitates were blotted against Tyro3 we also were able to detect it as demostrated by tnhe presence of its signature doublet (Fig. 5D lane 2). Given that the antibodies used against Tyro3 and Axl are specific for each of the receptors (see Fig. 1) these experiments suggest that Tyro3 and Axl closely interact.

## Discussion

In this study we explored the mitogenic properties of the receptor Tyro3 and its interactions with the related receptor Axl. We also investigated the potential signaling mechanisms mediating the mitogenic response and determined that the co-expression of Tyro3 and Axl influenced the relative activation of the MAPK and PI(3)K signaling pathways. Upon treatment with Gas6, expression of Tyro3 induced an increase in Axl phosphorylation and in turn, Tyro3 was phosphorylated in trans by Axl indicating a functional interaction between these TAM receptors.

Gas6 can elicit a proliferative response in several cell types including NIH-3T3 cells, VSMCs, [32], [33], cardiac fibroblasts [34], mesangial cells [35], prostate cells [36] and Schwann cells [37] with the mitogenic effects ascribed primarily to Axl and Mer. The precise role of each of the TAMs has been difficult to assess as in most cases the presence and activation state of each of the 3 receptors was not evaluated. In human Schwann cells that express both Axl and Tyro3, Gas6 induced robust cell proliferation and activation of ERK1/2. Axl may have mitogenic potential in its own right, since mesangial cells that express Axl but not Tyro3 show a proliferative response to Gas6 in a STAT3 phosphorylation-dependent manner. The interpretation of this result is complicated by the potential presence of eyk/Mer, which can signal through STAT3 to induce cellular transformation [35], [46], [47], [48].

In Rat2 cells, the introduction of Tyro3 increased Gas6-mediated proliferation, which appeared to be partially mediated by the MAPK pathway and not by the PI(3)K pathway. Tyro3 overexpression caused a significant increase of ERK1/2 phosphorylation, which was not observed when Axl was overexpressed. Tyro3 has been shown to activate MAPK signaling in other systems including cortical neurons [22] and osteoclasts [49].

In contrast, the overexpression of Axl failed to induce a significant increase in ERK1/2 phosphorylation but instead led to a robust activation of AKT as has been previously reported [16], [25]. Although Axl has been previously shown to activate ERK1/2, the TAM receptor composition of those cells was not defined [31], [34].

A recent study in which Axl expression was enhanced over that of Tyro3 and Mer showed a modest suppression of cell proliferation in prostate carcinoma cell lines and this reduction could in turn be blocked by ERK1/2 inhibitors [50] a finding that differed from an earlier study [36]. Recently, Tyro3 was detected in a cell line [20] previously used to demonstrate that Axl could induce both ERK1/2 and PI(3)K activation [51], underscoring the need to evaluate the specific complement of TAMs expressed. Consistent with our observations, others studies have failed to observe ERK1/2 activation and mitogenesis in response to Axl activation [52].

The inability of PI(3)K inhibitors to block proliferation suggested that this signaling pathway did not play a significant role in the Gas6-mediated proliferation in the Rat2/T3V5 cell line. The introduction of Tyro3 into the Axl-only expressing cell line reduced the baseline level of activation of several members of the PI(3)K signaling pathway despite increases in Axl phosphorylation. Tyro3 and Axl have both been previously shown to activate the PI(3)K signaling pathway [16], [25]. Axl has two consensus binding sites for p85 [52], [53] and one of these can bind both Grb2 and p85 in intact cells [54]. Tyro3 also has a consensus p85 binding site [29] and has been shown to activate AKT [22], [23], mTOR and p70S6 kinase in cortical neurons [22]. What are the mechanisms that could account for a decrease in PI(3)K signaling upon Tyro3 expression? One possibility is that receptor cross-phosphorylation results in a pattern of tyrosine phosphorylation that is unique to the presence of the Axl/Tyro3 receptor pair leading to a decrease in the association of p85 with one or more of its binding sites in Tyro3 or Axl. Unique phosphorylation patterns have been documented for RPTK heterodimers including members of the PDGF family [55]. Alternatively, the Tyro3/Axl pairing could result in the association or activation of a molecule that antagonizes the function of PI(3)K. In this context it is of interest to note that Axl can bind the phosphatase C1-TEN, [56] that antagonizes PI(3)K signaling [57].

Receptor heterodimerization and functional cooperativity among the TAMs has been predicted based on functional comparisons using single and multiple TAM knockout animals [18], [19], [20], [40], [41], [42], [58]. We tested this hypothesis based on the observation that transfection of Rat2 cells Tyro3 resulted in a significant increase in Axl phosphorylation, the extent which depended upon the amount of Tyro3 present. The increased Axl phosphorylation occurred in the absence of any changes in the level of Axl expression. The increase in Axl phosphorylation required Tyro3 activity as this increase was not detected when a kinase inactive form of this receptor (kdTyro3) was introduced. This supports the concept that Tyro3 either directly or indirectly phosphorylates Axl. In a reciprocal experiment, the overexpression of Axl resulted in the phosphorylation of kdTyro3 indicating that Axl can directly or indirectly phosphorylate Tyro3. These observations are consistent with two reports showing that TAM receptor phosphorylation appears to be interdependent. In one study, Gas6 failed to activate Axl in platelets isolated from *tyro3^−/−^* mice, showing that Tyro3 was required for Gas6 induced Axl phosphorylation [42]. A second study showed that macrophages utilize Mer for phagocytosis of apoptotic cells, but a second TAM seems to be required for full activation of Mer since in *axl^−/−/^tyro3* ^−/−^ animals the phosphorylation of Mer is significantly reduced when challenged with apoptotic cells [41].

Our studies suggest that the TAM receptors Axl and Tyro3 are capable of cross-phosphorylation which may be either a direct or indirect process. The co-immunoprecipitation findings reveal that Tyro3 and Axl are closely associated. Co-precipitation of Tyro3 and Axl has also been observed in the GnRH cell line NLT [20]. It remains to be determined if this association is the result of direct heterodimerization between Tyro3 and Axl as has been observed for several RPTK families including the ErbB [59], PDGF [45], and VEGFR [60] families.

These results suggest that changes in the expression levels of TAM receptors, such as those observed during development or malignant transformation, have significant implications for cell function. It will be important to determine if the changes in Tyro3 and Axl phosphorylation occur as the result of receptor heterodimerization which is often acompanied by changes in ligand binding preferences and receptor affinities that can influence the signaling properties of a cell. The demonstration that Tyro3 and Axl can cross-talk sheds new light onto the mechanisms underlying *in vivo* observations obtained using TAM knockout animals suggesting functional interaction among members of this receptor family [18], [41], [42]. Our studies underscore the need to identify the TAM receptor repertoire when characterizing the signaling properties underlying functional changes mediated by TAM ligands.

## Materials and Methods

### Ethics Statement

All procedures involving animal derived tissue samples and the production of anti-sera adhered to the guidelines established and approved by the NIH Guide for the Care and Use of Laboratory Animals and the Animal Care and Use Protocol #08-078 approved by the Indiana University Institutional Animal Care and Use Committee.

### Antibodies and Reagents

Recombinant human (h)Gas6 and hAxl-FCs was provided by Amgen Inc. (Thousand Oaks, CA). Antibodies were obtained from: anti-Axl (AF154) and anti-Mer (AF591) from R&D Systems (Minneapolis MN), anti-Mer antibody (MKT-101AP) from FabGennix International Inc, (Frisco, TX); anti-phospho (p) ERK1/2 (9101), anti-pp70S6kinase (9205), anti-p70S6kinase (9202), anti-pAKT (9271), anti-AKT (9272), anti-mTOR (2972), and anti-pmTOR (2971) from Cell Signaling Technology (Danvers, MA); anti-MAPK (anti-ERK1/2 M-5670), from Sigma-Aldrich (St Louis, MO) anti-phospho-tyrosine (ptyr) PY99 (sc-7020) and PY20 (sc-508) from Santa Cruz Biotechnology (Santa Cruz, CA); anti-glyceraldehydephosphodehydrogenase (GAPDH) (CA92590) from Chemicon (Temecula, CA); horse-radish peroxidase (HRP) conjugated goat anti-rabbit IgG and goat anti-mouse IgG from Thermo Scientific Inc, Pierce, (Rockford IL). Polyclonal rabbit antibodies recognizing the second fibronectin type III repeat of Tyro3, anti-FN2, were affinity purified in house from serum 2782 as previously described [43]. Polyclonal rabbit anti-hAxl was prepared in house as described below.

Full-length murine pCMVSport6.1-Axl cDNA was purchased from Open Biosystems (Thermo Scientific) (clone # 6313662).

### Generation and Purification of Axl Antibodies

Axl antisera were raised in rabbits (Myrtle’s Rabbitry Thompson’s Station, TN) by using hAxl-Fc receptor bodies [9] (AMGEN). The immunization regime was previously described in Prieto *et al* 2000 [43] and followed guidelines established by the NIH Guide for the Care and Use of Laboratory Animals and a protocol approved by the Indiana University Institutional Animal Care and Use Committee. Anti-Axl antibodies were affinity-purified by sequentially passing the anti-Axl serum through an IgG -Sepharose 4B column (Amersham Biosciences) for removal of anti-Fc antibodies, followed by incubation of the eluate with hAxl-Fc Sepharose-4B beads. The purified antibodies were characterized as previously described for the Tyro3 antibodies [43] and their selectivity for Axl shown in Fig. 1A in the Results section.

### Tyro3 and Axl cDNA Constructs

A full-length V5-tagged Tyro3 construct was generated by cloning an EcoRI/XhoI fragment from clone 18A [27] into pCDNA3.1/V5-HisA vector (Invitrogen, Carlsbad, CA). A segment between the BstEII site and the Tyro3 stop codon was amplified by PCR using primers designed to clone it in frame to the XbaI site in the vector upstream of the V5 epitope tag. The “kinase dead” (kd)Tyro3 construct was generated using the method described by Nelson *et al* 1989 [61]. Two point mutations were introduced, the first changing lysine 535 to methionine (K535M) (A to T at bp1838) and the second introducing an EcoRV site (C to T at bp 1855) (accession # X78103). The NotI/BstEII fragment in wild-type Tyro3 was replaced by the one containing the K-M mutation and the EcoRV site.

### Cell Culture and Gas6 Activation

The Rat2 untransfected cells (ATCC, Manassas, VA) were used to generate the Rat2/T3V5 cells lines as described under “**Cell Transfection**”. The cells were grown in DMEM, 2 mM L-glutamine, penicillin (100 U/ml) and streptomycin (100 µg/ml) (all from Invitrogen) and 10% fetal calf serum (FCS) (Omega Scientific, Tarzana, CA). For Gas6 activation experiments the cells were serum starved in 0.5% FCS for 48 hrs and replaced with 0% DMEM for 4 hrs before stimulation. For activation, Gas6 was added (0, 50 or 350 ng/ml) for 10–20 min at 37°C. When the activation was performed in the presence of the inhibitors wortmannin (1.5 µM), LY294002 (5.5 µM) (both from Sigma-Aldrich), or U0126, (1.0 µM and 10 µM) (Cell Signaling Technologies) they were added 45 min prior to addition of Gas6. Dimethylsufoxide (DMSO) (Sigma Aldrich) was used as vehicle only control. After activation, cell harvesting and lysis were carried out as previously described [22].

### Immunoprecipitation and Western Blotting

For Tyro3, Axl and Mer immunoprecipitation (IPs) from cell and tissue extracts, lysates containing equal amounts of protein were incubated overnight at 4°C with primary antibodies. We used 0.8 µg/ml of affinity-purified anti-FN2 Tyro3 antibody [43], 2.5 µg/ml of anti-Axl # AF154, or 2.5 µg/ml of anti-Mer #AF591 antibody. For IPs using mouse monoclonal anti-Axl and anti-Mer antibodies we used 30 µl of a 1∶1 Protein G bead/lysis buffer slurry or for rabbit anti-Tyro3 antibodies 30 µl of a 1∶1 Protein A bead/lysis buffer slurry (GE Healthcare). Immunoprecipitation and Western blotting was performed using 8% or 4–20% Tris-glycine gels (Invitrogen) as previously described [22]. The antibodies used for the Western blot analyses were: rabbit anti-Tyro3 serum #5424 (1∶3,500 dilution) [27], rabbit anti-Axl affinity purified (1∶3,500 dilution), rabbit anti-Mer MKT-101AP (1∶2,000 dilution), a 1∶1 mixture of anti-pTyr PY99 and PY20 (1∶3,500 dilution), anti-ERK1/2 (MAPK) (1∶5,000), anti-pERK1/2, pAKT, AKT, pp70S6kinase, p70S6kinase, pmTOR, mTOR antibodies were all used at (1∶1,000 dilution), and anti-GAPDH (1∶3,500 dilution). Due to variability in the efficiency of stripping the phosphor-specific antibodies from the blots shown in Fig. 3A and B the samples were reloaded and blotted with antibodies recognizing all forms of the proteins in separate gels with the appropriate controls to ensure equal loading, these data are shown in Fig. 3C and D.

### Deglycosylation

Rat2/T3V5 cell and brain detergent extracts lacking SDS, corresponding to 10 µg of protein were treated overnight at 37°C with 500 units of N-glycosidase F (PNGase F) using the PNGase F kit from New England Biolabs (NEB, Beverly, MA) according to the manufacturer’s instructions. The reaction was stopped by the addition of Laemmli sample buffer, denatured for 3 min at 98°C, and analyzed by SDS-PAGE as described in **“Immunoprecipitation and Western Blotting”**.

### Cell Proliferation Assay

Rat2 cells and Rat/T3V5 cells (25,000 cells/well) were seeded in 24 well plates for 24 hrs. The media was changed to 0.5% serum containing 0–500 ng/ml of Gas6. Pharmacological inhibitors were added 45 min prior to Gas6 addition when used. They included the PI(3)K inhibitors wortmannin (1.5 µM) and LY294002 (5.5 µM) the MEK inhibitor U0126, (5.0 µM) and DMSO as control. For the time-course experiments the cells were incubated in Gas6 for 0, 24, 48, and 72 hrs with 250 ng/ml of Gas6. For pathway-inhibitor experiments the incubation period was 72 hrs with 250 ng/ml of Gas6.

Changes in proliferation were determined using the WST-8, (2-(2-methoxy-4-nitrophenyl)-3-(4-nitrophenyl)-5-(2,4-disulfophenyl)-2H-tetrazolium, monosodium salt) (Dojindo, Gaithersburg, MD) reagent as described by the manufacturers. The optical density of a total of 4 wells per condition was averaged in each experiment, and the results of three independent experiments were averaged using the 2-sample t-test.

### Cell Transfection

Rat2 cells were plated in 6 well plates at a density of 0.4×10^6^ cells/well and were transfected with pcDNA3.1/Tyro3V5-HisA vector (Invitrogen) encoding full-length mouse Tyro3, or pCDNA3.1/laczV5-HisA using Lipofectamine Plus following manufacturer instructions (Invitrogen). Neomycin resistant clones were selected with 600 µg/ml G418 (Omega Scientific) and tested for Tyro3 expression as previously described [43]. Our efforts to perform signaling and proliferation experiments in Rat2 cell lines overexpressing Axl after selection of permanently transfected cells were not feasible. Despite considerable effort, we were unable to obtain lines that expressed Axl at levels over those observed in untransfected cells. We tested 3 different Axl constructs, utilized multiple transfection protocols and selected and screened over 200 antibiotic resistant lines.

For transient transfection experiments with 2 plasmids, Rat2 cells were plated in 6 well plates at a density of 0.4×10^6^ cells/well and transfected as described above with 2 µg of wtTyro3/V5, kdTyro3/V5 or lacZ/V5 or by combining these vectors with 4 µg of Axl cDNA. The cells were grown for 24 hrs before changing the media overnight to 0.5% serum, and again to no serum 4 hours before the activation experiments were performed.
